# Prevalence, predictors and triggers of migraine headache among medical students and interns in King Abdulaziz University, Jeddah, Saudi Arabia

**DOI:** 10.12669/pjms.332.12139

**Published:** 2017

**Authors:** Nahla Khamis Ibrahim, Afnan Khalid Alotaibi, Abrar Mansour Alhazmi, Rawan Zaher Alshehri, Rawan Nabeel Saimaldaher, Maradi Abdulkader Murad

**Affiliations:** 1 Nahla Khamis Ibrahim, Professor at Family & Community Medicine Department, King Abdulaziz University, Jeddah, Saudi Arabia. Professor, Epidemiology Department, HIPH, Alexandria University; 2 Afnan Khalid Alotaibi, Sixth Year Medical Students at King Abdulaziz University, Jeddah, Saudi Arabia; 3 Abrar Mansour Alhazmi, Sixth Year Medical Students at King Abdulaziz University, Jeddah, Saudi Arabia; 4 Rawan Zaher Alshehri, Sixth Year Medical Students at King Abdulaziz University, Jeddah, Saudi Arabia; 5 Rawan Nabeel Saimaldaher, Sixth Year Medical Students at King Abdulaziz University, Jeddah, Saudi Arabia; 6 Maradi Abdulkader Murad, Sixth Year Medical Students at King Abdulaziz University, Jeddah, Saudi Arabia

**Keywords:** Headache, Medical students, Migraine, Predictors, Prevalence, Outcomes, Triggers

## Abstract

**Objectives:**

To determine the prevalence, predictors, triggers and educational outcome of migraine among medical students and interns in King Abdulaziz University, Jeddah, Saudi Arabia.

**Methods:**

A cross-sectional study was completed among 566 participants selected through a multistage stratified random sample method. A validated, confidential, self-administered data collection sheet was utilized. It contained ID Migraine test™, Numeric Pain Rating Scale (NPRS). Questions about possible predictors, triggers and impact of migraine were asked. Descriptive, inferential statistics and multiple logistic regression analysis were conducted.

**Results:**

More than one-half (54.9%) of the participants had ≥ 2 headache attacks during the three months preceded the study. The prevalence of migraine was 26.3%, and 41.6% of the cases suffered from severe pain. The main migraine predictors were Functional Gastrointestinal Disorders (FGIDs), family history of migraine, female gender, and enrollment in the second academic year. Exam stress and sleep disturbances were the commonest triggers. The majority of the participants reported that their educational performance and ability to attend sessions were affected during migraine attacks.

**Conclusion:**

A relatively high prevalence of migraine was seen among our participants. FGIDs, gender and academic year were the predictors. Screening and management of migraine among medical students are required. Conduction of relaxation programs and stress management courses are also recommended.

## INTRODUCTION

Nowadays, headache has been considered as one of the top global disabling medical conditions.^1^ Migraine is an important type of headache, and one of the chronic multifaceted neuro-inflammatory disorders.^2^ It is characterized by recurrent throbbing headache pain that typically affects one side of the head, and is often accompanied by nausea and disturbed vision. Migraine headache accounts for 1.4% of all neurological and mental disorders.^2^ It was reported that the estimated lifetime prevalence of migraine ranged 12%-18%.^3^

Migraine is considered an important health problem among university students. This is due to its high prevalence, associated morbidities, disability and decreasing academic performance.^3^ Medical student are usually working hard and requiring constant concentration and studying, which may cause much stresses and sleep disturbances, subjected to high stressful conditions.^4^

There is inadequate number of recent studies done about migraine headache among medical students in Jeddah, Saudi Arabia. Hence, such study was required. The objective of the study was to determine the prevalence, predictors, triggers and educational outcome of migraine headache among medical students and interns in King Abdulaziz University (KAU) in Jeddah, Saudi Arabia.

## METHODS

A cross sectional study was conducted during the educational year 2014/2015. The study population included medical students who completed the freshman year (2^nd^ – 6^th^ year), and interns at KAU. A multistage stratified random sample method was used. Stratification put into account the educational level and gender. The sample size was calculated according to the following pre-established formula:^5^

The calculated sample size to achieve a precision of ± 0.04%, at a 95% Confidence Interval (CI) was 600 participants. A validated, confidential, self-administered data collection sheet was used. The face and content validity of the instrument was assessed by two experts. Internal consistency reliability was assessed by Cronbach’s alpha test and found to be 0.8. The data collection sheet asked about personal, socio-demographic data, habits, Grade Point Average (GPA), and history of ≥ 2 general headache attacks during three months preceded the study. History of chronic diseases, Functional Gastrointestinal Disorders (FGIDs) other than Irritable Bowel Syndrome (IBS), and IBS were asked. Anxiety and depression were assessed. Weight and height were determined.

The English version of ID Migraine™ test (with good sensitivity and specificity)^6^ was used. The detected migraineurs were asked about migraine regarding the age onset, frequency, duration of attacks, associated symptoms, triggers, etc. In addition, the Numeric Pain Rating Scale (NPRS) (7) was used to assess severity of migraine during attacks. For females, the effect of menstrual period on migraine was asked. Drugs utilized for relieving migraine headache was determined. Educational outcomes of migraine were assessed.

### Statistical analysis

The data was analyzed using SPSS (21). Body Mass Index (BMI) was calculated. The severity of migraine pain was classified by NPRS into mild, moderate and severe degrees.^7^ Descriptive statistics were done. Pearson’s Chi-square (X^2^), Odds Ratios (ORs) and 95% CIs were calculated. A multiple logistic regression analysis model was done. All P-values < 0.05 were considered statistically significant.

### Ethical statement

The research was conformed to Helsinki Declaration. The protocol of the study was approved by the Institutional Review Board (IRB) of King Abdulaziz University Hospital (KAUH), with a Reference Number of 334-14. A written informed consent was taken from each student accepted participant. Administrative approvals were also taken.

## RESULTS

Out of 600 invited medical students and interns, 566 completed the questionnaire (acceptance rate= 94.3%). Their mean age was 21.5 ± 1.6 years. The prevalence of having ≥ 2 headache episodes during the three months preceded study was 54.9%. Furthermore, the prevalence of migraine headache was 26.3% (47.9% of all types of headache). The mean age of start of migraine attacks was 16.9±3.6 years, and the mean number of attacks was 4.6± 1.5 per month. About one-third (34.8%) of female sufferers reported that their migraine headache is affected by menstrual cycle. NPRS revealed that 14.8%, 43.6% and 41.6% of migraineurs suffered from mild, moderate and severe degrees of pain, respectively.

The prevalence of migraine among female (33.2%) was much higher than males (15.5%), with a highly statistical significant difference (X^2^ = 21.93, p < 0.001). Table-I shows that students who enrolled in the second academic year had much higher prevalence of migraine headache compared to others (OR= 2.24; 95% CI: 1.39-3.59). Furthermore, participants with family history of migraine were 3.64 times more prone to have it compared to others (OR=3.64; 95% CI: 2.45-5.43). The prevalence of migraine was slightly higher among smokers and the better achiever students compared to others. However, there is no statistical difference (p > 0.05).

**Table-I:** Relationship between migraine headache and personal, socio-demographic characteristics and characteristics of migraineurs in King Abdulaziz University.

*Variables*	*Migraine*				*X^2^*	*p*	*OR*	*CI*

	*Yes (n=149)*		*No (n=417)*	

	*No.*	*%*	*No.*	*%*
***Gender***								
Male	34	15.5	186	84.5	21.93	0.000	0.37	0.24 - 0.56
Female	115	33.2	231	66.8
***Age***								
>20	63	24	199	76	1.31	0.253	1.25	0.85 - 1.82
<=20	86	28.3	218	71.7
***Academic year***								
Second year	63	40.9	52	59.1	11.43	0.001	2.24	1.39 - 3.59
Other years	113	23.6	365	76.4
***Marital status***								
Single	147	27	397	73	3.51*	0.061	3.70	0.86- 16.04
Married	2	9.1	20	90.9
***Family history of migraine***								
Yes	73	45.6	87	54.4	42.84	0.000	3.64	2.45 -5.43
No	76	18.7	330	81.3
***Smoking***								
Yes	17	27.4	45	72.6	0.43	0.83	1.06	0.58 – 1.92
No	132	26.2	372	73.8
BMI*								
Normal	100	27.2	267	72.8	0.29	0.59	1.12	0.75 – 1.66
Overweight& obese	49	25.1	146	74.9
***Number of studying hours/week***								
≥ 14hours/week	88	27.8	228	72.2	0.87	0.36	1.20	0.82 – 1.75
<14hours/week	61	24.4	189	75.6
***GPA***								
≥ 4.5 (≥90%)	79	29.7	187	70.3	2.94	0.08	1.38	0.95 – 2.02
< 4.5 (< 90%)	70	23.3	230	76.7				

N.B.

* 4 students (non- migraineurs) didn’t have recent measurements of their weight and height.

Migraine was associated with presence of chronic diseases (OR= 2.38; 95% CI: 1.52 - 3.74). Table-II Participants with FGIDs (other than IBS) were approximately 4.5 times more prone to had migraine headache compared to others (OR= 4.48; 95% CI: 1.79 - 11.18). Similarly, migraine headache was higher between IBS sufferers (OR= 2.56; 95% CI: 1.30 - 5.02). The prevalence of migraine was higher among participants complaining from anxiety and depression (p > 0.05).

**Table-II:** Relationship between migraine headache and presence of chronic and psychological conditions among participants in King Abdulaziz University.

*Variables*	*Migraine*				*X^2^*	*p*	*OR*	*CI*

	*Yes*		*No*	

	*No.*	*%*	*No.*	*%*
***Chronic diseases***								
Yes	42	41.6	59	58.4	14.75	0.000	2.38	1.52 - 3.74
No	107	23.0	358	77.0
***Functional gastrointestinal disorders (other than IBS)***								
Yes	12	60.0	8	40.0	12.12	0.000	4.48	1.79 - 11.18
No	137	25.1	409	74.9
***Irritable bowel syndrome***								
Yes	17	45.9	20	54.1	7.86	0.005	2.56	1.30 - 5.02
No	132	25.0	397	75.0
***Endocrine diseases***								
Yes	4	44.4	5	55.6	1.54*	0.21	2.27	0.60- 8.58
No	145	26.0	412	74.0
***Bronchial asthma***								
Yes	5	37.5	9	64.5	0.65	0.14	1.57	0.52 – 4.77
No	144	26.1	408	73.9
***Anxiety***								
Morbid anxiety	15	60.0	10	40.0	15.29	0.000	4.56	1.99 – 10.38
No morbid anxiety	134	24.8	407	75.2
***Depression***								
Morbid depression	9	60.0	6	40.0	9.01	0.003	4.40	1.54 – 12.59
No depression	140	25.4	411	74.6				

Controlling confounding factors reveals that FGIDs was the first predictor of migraine headache (aOR= 3.30; 95% CI: 1.07-10.11), followed by family history of migraine (aOR=3.06; 95% CI: 2.03-4.63), female gender (aOR= 2.74; 95% CI: 1.73-4.33), and enrollment in the second year of university education. Table-III.

**Table-III:** Logistic regression analysis of the predictors of migraine headache among medical students in King Abdulaziz University

*Variable*	*Beta*	**P**	*aOR*	*95% CI*
Functional gastrointestinal disorders	1.19	0.03	3.30	1.07-10.11
Family history of migraine	1.12	0.000	3.06	2.03-4.63
Female gender	1.01	0.000	2.74	1.73-4.33
Second year medical students	0.92	0.001	2.51	1.49-4.22
Constant	-8.869

aOR: Adjusted Odds Ratio

The commonest reported manifestations accompanying migraine headache were difficulty in concentration (80.5%), photophobia (73.5%), light-headed feeling (53.0%) and nausea (52.3%). Table-IV. Results showed that the most frequently reported migraine triggers were exam stress (82.6%), sleep disturbance (79.9%), emotional stress (73.2%), noise (71.1%), bright lights (69.1%), extended reading hours (64.4%) and anxiety (52.3%). Hunger, depression, caffeine withdrawal, physical activity, smoking and food were reported triggers by 41.6%, 40.9%, 34.9%, 24.2%, 16.1% and 14.1% of them, respectively.

Regarding drugs, 16.8% of the migraineurs utilized prescribed medications, 72.5% took Over-the-Counter (OTC) analgesics and 10.7% didn’t use any medications for migraine. Paracetamol was the most commonly (66.7%) used medication group, followed by both Paracetamol and Non-steroidal Anti-Inflammatory Drugs (NSAIDs) together (14.8%) and NSAIDs alone (6.5%), and other drugs.

Regarding educational outcome of migraine, the majority of migraineurs reported that their educational performance (83.9%) and ability to attend classes (78.2%) were reduced during migraine attacks.

## DISCUSSION

The current study illustrated that more than one-half of the participants suffered from ≥ 2 headache attacks during the three months preceded the study. This coincides with other study done among medical students from Isfahan, Iran.^8^ Results from an Indian study showed that migraine constituted 42% of all types of headache,^9^ which agrees with our findings.

The prevalence of migraine headache among our participants was 26.3%, which agrees with recent similar studies from Kuwait,^10^ USA^11^ and India.^9^ On the other hand, much lower rates were reported from other studies done in Iran,^8^,^12^ Turkey^13^ and Nigeria.^14^ On the other hand, a higher prevalence was reported from Peshawar, Pakistan.^15^ The causes of such discrepancies could be attributed to cultural differences between countries, difference in the time of conduction of their studies, the amount of educational stress and the instrument used for diagnosis of migraine. In the current work, the mean number of migraine attacks was 4.6 attacks / month, which is in line with the results from Kuwait^10^ and Pakistan.^15^

Our findings revealed that presence of migraine headache was associated with female gender. This was supported by results from many other studies.^10^,^11^,^16^ In addition, about one-third of female migraineurs in our study reported that migraine was affected by their menstrual cycle, which agrees with the results from Croatia.^16^ Endogenous sex steroid hormones may have a relevant role in explaining of such findings.^17^

The second year students in our study reported significantly higher rate of migraine compared to others. This can be explained by numerous stressors that face medical students during the first medical education year (after freshman year). However, our results disagree with the Croatian study.^16^ The cause of discrepancy may be attributed to cultural differences or the amount of faced stresses.

Results of the present study revealed presence of an association between migraine headache and the family history of it. This finding agrees also with the results of other studies.^8^,^14^ FGIDs was the first predictor of migraine in the current study and this is in line with other studies.^18^,^19^ The possible physiological pathways of migraine may be associated with the brain-gut axis, neuro-immunity, and neuro-endocrine interactions.^18^

Our results showed that stresses, and sleep disturbance were the commonest reported triggers, which agree with results from India^9^ and Kuwait.^10^ Furthermore, smoking was reported as a trigger of migraine among approximately 16% of migraineurs which is in line with results from Spain.^20^

The commonest accompanying symptoms of migraine in our study were difficulty in concentration and photophobia. Similarly, results from the Indian study reported that photophobia was the commonest manifestation.^9^

Paracetamol was the most frequently used analgesic for migraine in the current study. On the other hand NSAIDs were the most commonly used by US students.^11^ This discrepancy may be due to widespread use of Paracetamol in Saudi Arabia.

The present study showed that educational performance and the ability to attend educational classes were affected to a certain degree among the majority of migraineurs during migraine attacks. These findings are in line with findings from a study from the USA.^11^

## CONCLUSION

A relatively high prevalence of headache and migraine was apparent among participants in the current study. FGIDs, family history of migraine, female gender, and the enrollment in the second academic year were migraine predictors. Stresses and sleep disturbances were the most frequently reported triggers. The majority of participants reported reduced ability of attending educational session and educational performance during migraine attacks. Conduction of similar studies among all university students is needed. Screening programs for migraine and FGIDs are needed, with referral and management of the diagnosed cases among medical students. Relaxation programs, stress management courses and providing good guidance to avoid stress are required. Special educational programs are needed to raise awareness about migraine.
